# Spatial Analysis of the Distribution, Risk Factors and Access to Medical Resources of Patients with Hepatitis B in Shenzhen, China

**DOI:** 10.3390/ijerph111111505

**Published:** 2014-11-07

**Authors:** Yuliang Xi, Fu Ren, Shi Liang, Jinghua Zhang, De-Nan Lin

**Affiliations:** 1 School of Resources and Environmental Science, Wuhan University, Wuhan 430079, China; E-Mails: yuliangwhu@163.com (Y.X.); zhangjinghuawh@163.com (J.Z.); 2 Key Laboratory of Geographic Information Systems, Ministry of Education, Wuhan University, Luoyu Road 129, Wuhan 430079, China; 3 Shenzhen Center for Health Information, Renmin Road North 2210, Luohu District, Shenzhen 518001, China; E-Mail: ldn308@163.com

**Keywords:** spatial epidemiology, spatial analysis, hepatitis B, Shenzhen

## Abstract

Considering the high morbidity of hepatitis B in China, many epidemiological studies based on classic medical statistical analysis have been started but lack spatial information. However, spatial information such as the spatial distribution, autocorrelation and risk factors of the disease is of great help in studying patients with hepatitis B. This study examined 2851 cases of hepatitis B that were hospitalized in Shenzhen in 2010 and studied the spatial distribution, risk factors and spatial access to health services using spatial interpolation, Pearson correlation analysis and the improved two-step floating catchment area method. The results showed that the spatial distribution of hepatitis B, along with risk factors as well as spatial access to the regional medical resources, was uneven and mainly concentrated in the south and southwest of Shenzhen in 2010. In addition, the distribution characteristics of hepatitis B revealed a positive correlation between four types of service establishments and risk factors for the disease. The Pearson correlation coefficients are 0.566, 0.515, 0.626, 0.538 corresponding to bath centres, beauty salons, massage parlours and pedicure parlours (*p* < 0.05). Additionally, the allocation of medical resources for hepatitis B is adequate, as most patients could be treated at nearby hospitals.

## 1. Introduction

The hepatitis B virus (HBV) is one of the most common viral infections in the world, leading to many deaths each year from associated liver diseases [1,2]. HBV is also an important public health issue in China [2], as national surveys conducted since the 1970s have repeatedly showed high morbidity from HBV infections in the general population [3,4]. To address this challenge, many scholars now to study the disease using different approaches (e.g., pathology, immunology and epidemiology), but none of these approaches incorporate spatial information [5]. With the development of Geographic Information System (GIS) technology and its applications in epidemiology [6], it has become clear the spatial information has great value in Epidemiology. Spatial epidemiology is a branch of epidemiology that uses spatial information to extend the analysis of disease distribution and risk factors. This information serves as a powerful analytical tool for epidemiological studies and will likely show greater significance in the future [7]. Our team have done some works on spatial epidemiology [8,9,10].

Using geographic information systems and spatial analysis technology, spatial epidemiology attempts to describe and analyze human diseases, including the spatial distribution characteristics of health and hygiene events as well as the regularity of changes and developments. Spatial epidemiology also explores the decisive elements that can impact public health and provides strategies and measures for disease prevention and cure and health promotion and hygiene services [7,11]. Research on this subject mainly utilizes four elements: disease mapping, risk assessment in relation to a point or line source, disease clustering analysis and geographical correlation studies [11]. Disease mapping is the most important tool in spatial epidemiology and is used to describe and evaluate disease area distribution [7]. The most typical example of disease mapping is the epidemiological investigation of a cholera outbreak caused by street water pollution in London in 1854 [12,13]. The assessment of risk factors is similar to geographical correlation studies in that they both focus on studying cause of geographical phenomenon. Moreover, analysis of disease clustering uses statistical methods to identify disease spatial aggregation, thus providing corresponding methods to search for etiologic clues [7,14,15].

Among the four study elements, disease mapping and disease clustering analysis are relatively mature, and typical methods and models, such as spatial interpolation and spatial scan statistics are commonly used [16,17,18]. However, exploring the risk factors for disease is complex and difficult, especially for diseases in which it is difficult to obtain patient exposure information, and data involving patients’ privacy [7]; this challenge represents a weak link in epidemiological studies. Compared with other infectious diseases such as schistosomiasis [19,20], most respiratory system diseases [21] and cardiovascular diseases [8], the spatial risk factors for hepatitis B are less obvious, and there have been few studies on this aspect. In view of this problem, this study sought to explore the spatial risk factors for hepatitis B and to discover the relationship between these risk factors and hepatitis B morbidity.

In addition, the prevention and control of disease is the main goal of spatial epidemiology, and one important research subject is spatial access to health services. Spatial access to health services refers to overcoming spatial obstacles to medical facilities [22,23]. Based on different application environments and requirements, many different research methods are used to measure spatial access. Among the numerous methods, the gravity model (also known as the potential model) and the two-step floating catchment area method are the most widely used [24,25]. However, both methods have flaws; the gravity model is somewhat abstract and difficult to understand, and the two-step floating catchment area method ignores the spatial access differences in demand points within the same search scope and the spatial access values in demand points outside of the search scope [26]. It is also difficult to set a reasonable search radius for each medical facility [27]. This current study used an improved two-step floating catchment area model to overcome these limitations. In particular this model considers the effect of distance decay by setting weighted distance values, and this improved method is also an effective method for setting a reasonable search radius for each medical facility. These two changes significantly improve the accuracy of measuring spatial access to health services.

## 2. Data and Methods

### 2.1. Study Area

Shenzhen is a coastal city located in the south of China, northeast of the Pearl River Estuary. This city is located south of the Tropic of Cancer, from 113°46′ to 114°37′ east longitude and between 22°27′ and 22°52′ north latitude, and the total area of Shenzhen is approximately 1952.84 km^2^ [27].

**Figure 1:**
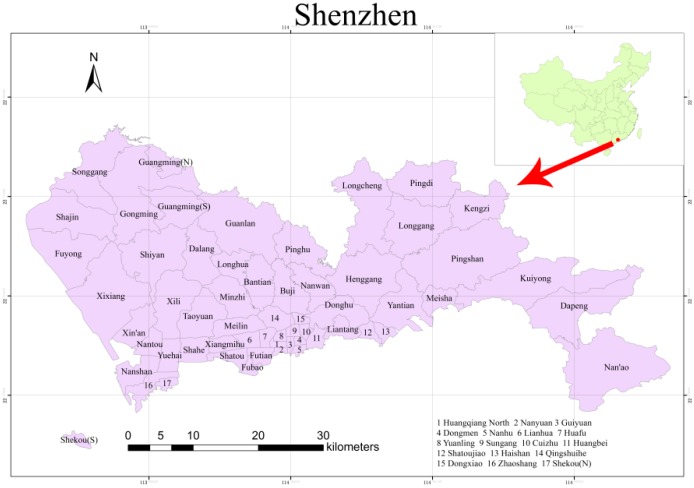
The study area.

Shenzhen is located at the border between subtropical monsoon and tropical marine climates with abundant rainfall and beautiful scenery. The average annual precipitation is approximately 1924.7 mm. With 230 km of coastline, Shenzhen is rich in marine resources such as excellent ports and abundant fisheries. Shenzhen was the first special economic zone in China. With its rapid economic development, Shenzhen occupies a position of importance in China. At present, there are a total of 10 districts and 57 sub-districts in Shenzhen. Figure 1 shows the location of Shenzhen in China and the names of its sub-districts.

### 2.2. Study Data

The study area for this paper was the whole of Shenzhen City, which includes 10 districts and 57 sub-districts. The study mainly collected geographic, demographic, and hepatitis B case data, along with some service facility and medical resource data:

1. Basic geographic data: Administrative data for the division of Shenzhen were obtained from the Urban Planning, Land and Resources Commission of the Shenzhen Municipality [9].
2. Demographic data: These data, for all 57 different sub-district administrative regions, were obtained from the 6th national population census [28].
3. Hepatitis B case data: Data from the Shenzhen Centre for Health Information (SCHI), an institute directly administered by the Health, Population and Family Planning Commission of Shenzhen Municipality, were obtained from hospitalized patients’ medical records including the patients’ home addresses, ages, sexes, *etc.* in 2010.
4. Medical facility data: These data were also obtained from the Shenzhen Centre for Health Information with addresses and service levels for most hospitals.
5. Service facility data: Certain service facilities may promote hepatitis B infection. Address data were obtained by searching electronic maps on the Internet.

### 2.3. Study Methods

This study mainly applied spatial interpolation (Kriging), spatial risk factors correlation analysis and analysis of spatial access to health services. To be pointed out that the Shenzhen Center for Health Information (SCHI), an institute directly administered by the Health, Population and Family Planning Commission of Shenzhen Municipality gave the permission of the research involving hepatitis B cases and approved this retrospective study. In this study, written informed consent was obtained by participants so we could not reveal their personal information to the public. In addition, the study was approved by the Wuhan University Institutional Ethnic Committee, and we must keep the patients’ information secret.

#### 2.3.1. Spatial Interpolation

Spatial interpolation is a process of intelligent guesswork in which the investigator attempts to make a reasonable estimate of the value of a continuous field in places where the field has not actually been measured [29]. This operation only makes sense from the continuous-field perspective. In addition, spatial interpolation can weaken the influence of administrative boundaries on the spatial distribution of certain attributes, and its methods include point and areal interpolation [30]. This paper adopted point interpolation, which is widely used in disease mapping to predict the disease morbidity in a specific area. Many methods can be used for spatial interpolation, such as inverse distance weighting, global polynomial, local polynomial, spline function, kriging, *etc.*

Among the various interpolation methods, kriging combines the advantages of other methods and also considers spatial variation factors, which are critical in the study of spatial epidemiology. Kriging is the best interpolation method, when the statistical data satisfies the hypothesis (either a normal distribution and second-order stationary hypothesis or an intrinsic hypothesis) [31]. Consequently, it is possible to precisely describe the random space processes of illness.

Kriging uses variograms which are widely used to describe regionalised variables. A variogram is defined as a type of variance in regionalised variables and is important in Geostatistical analysis [32]. Under the conditions of either a second-order stationary hypothesis or an intrinsic hypothesis, any random *h* can be indicated by the following formula:

(1) $$\gamma (h)=\frac{1}{2}Var[Z(x)-Z(x+h)]=\frac{1}{2}E\{{\left[Z(x)-Z(x+h)\right]}^{2}\}$$

If the sample points are presented as a discrete distribution, then the formula is:

(2) $$\gamma^{*}(h)=\frac{1}{2N(h)}{\sum_{i=1}^{N(h)}\left[Z(x_{i})-Z(x_{i}+h)\right]}^{2}$$

where $\gamma^{*}(h)$ is the variogram and is the number of sample points with h spatial distance. $Z(x_{i})$ and $Z(x_{i}+h)$ represent the values of N(h) regionalized variables in $x_{i}$ and $x_{i+h}$.

**Figure 2:**
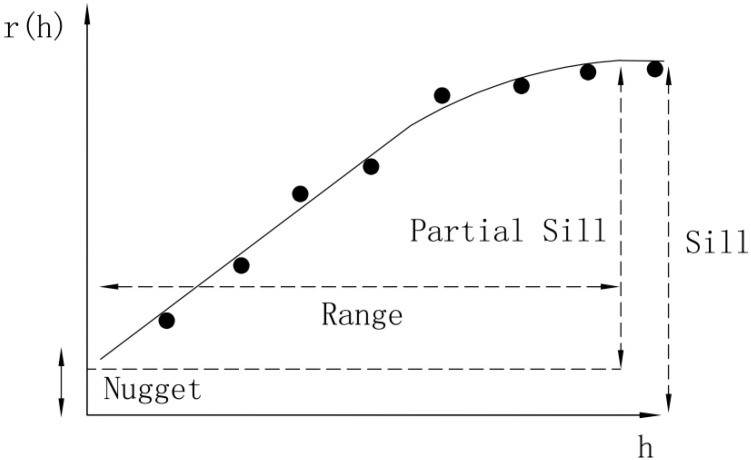
The variogram.

In the variogram model (Figure 2), spatial structures of geographical phenomena are mainly measured as nuggets, sills and ranges. The nugget is a random value reflected by spatial variability and measurement error.

When the distance between sample points is approximately equal to zero, random error and spatial variability lead to variations in the variogram values, which will not be zero at the origin points. Range refers to the sample points within the range of spatial dependence. If the distance between two points is greater than the range, the sample point value differences will be stable. A sill is a platform value when the range is unchanged, and the sill is used to measure variation in an entire system.

#### 2.3.2. Correlation Analysis of Spatial Risk Factors

Correlation analysis of spatial risk factors involves measuring and evaluating the correlations between a disease and its risk factors in a small area [7], and the general calculation for measuring risk factor and disease correlations is the Pearson correlation coefficient [33].

The Pearson correlation coefficient describes the degree of association between two variables. The coefficient is represented by the letter “*r*”, and the formula for the calculation is as follows:

(3) $$r=\frac{1}{n-1}\sum_{i=1}^{n}\left(\frac{X_{i}-\bar{X}}{\sigma_{X}})(\frac{Y_{i}-\bar{Y}}{\sigma_{Y}}\right)$$

where n is the sample number, $X_{i}$ and $Y_{i}$ are the observed values of two variables, $\bar{X}$ and $\bar{Y}$ are the averages of the two variables. R measures the degree of correlation between the two variables, $\sigma_{X}$ and $\sigma_{Y}$ represents the standard deviations of the two variables. The value of r is between −1 and 1. If *r* > 0, there is a positive correlation between the two variables, whereas if *r* < 0, there is a negative correlation between the variables. For larger absolute values of r, there is greater correlation between the two variables. If *r* = 0, there is no linear correlation between the two variables.

As shown in Table 1, the interpretation of the Pearson correlation coefficient put forward by some scholars is as follows [34,35]. However, it should be noted that all of these criteria are, to a certain extent, arbitrary rather than strict [35]. The interpretation of correlation coefficients depends on the specific application background and purpose. Because the subject of this study was affected by various and complicated social factors, the requirements for the calculation results were not strict; thus, the criteria in the table can be considered reliable and reasonable.

**Table 1:** Interpretation of the Pearson correlation coefficients.

Correlation	Pearson Correlation Coefficient (Positive)	Pearson Correlation Coefficient (Negative)
Irrelevant	0 to 0.09	−0.09 to 0
Weak correlation	0.1 to 0.3	−0.3 to −0.1
Medium correlation	0.3 to 0.5	−0.5 to −0.3
Strong correlation	0.5 to 1.0	−1.0 to −0.5

#### 2.3.3. Analysis of Spatial Access

Spatial access is an uncertain concept that depends on specific occasions or practical problems [27]. In terms of this paper, spatial access was defined as the extent of overcoming spatial obstacles. Based on two groups of combination, potential and realized and spatial and non-spatial, access can be divided into four types: potential spatial access, potential non-spatial access, realized spatial access, realized non-spatial access [36]. Realized access refers to the actual consumption of services, whereas potential access refers to the possibility of service consumption. In this study, potential spatial access to medical resources was the study focus, because this form of access can evaluate whether the regional distribution of medical resources is reasonable.

In the study of spatial access to medical resources, there are mainly three factors: spatial distribution and the supply of medical services; spatial distribution and the residential area; and the spatial relationship between the population and medical services [27].

There are many methods for evaluating the spatial access. Generally speaking, the gravity model and the two-step floating catchment area method are extensively used in this field. This paper used the improved two-step floating catchment area method, which is different from the enhanced two-step floating catchment area method (E2SFCA, Luo W) [37], to measure the potential spatial interaction between patients and hospitals across administrative regions.

The improved two-step floating catchment area method is based on the method proposed by Radke and Mu but includes two improvements. First, the improved method considers the effect of distance decay in the search scope, thus overcoming the shortcoming that all of the demand points have the same access within the search scope. Second, the improved method offers an effective way to set the search radius for each hospital.

The improved two-step floating catchment area method includes the following two main steps, which are further elaborated:

(1) Setting the search radius value and calculating the ratio of supply and demand.

(a) The search radius value can be set by the service level and capacity of each hospital. Hospitals are divided into three levels, with higher service levels, indicating a greater search radius value.
(b) To set a more accurate hospital service radius, a group gradient search radius value can be set at the same hospital level. Each level has three search radius values.
(c) Based on the requirements of the hospital, which mainly depend on the number of beds and the number of health technical personnel, each hospital’s service capacity can be calculated.

In sum, the hospital’s supply and demand ratio $R_{jl}$ can be indicated by the following formula:

(4) $$R_{jl}=\frac{S_{jl}}{\sum_{k\in \{d_{kj}\in D_{r}\}}P_{k}}(l=A,B,C)$$

where l is the hospital level, j is the number of hospitals at that same level, k is the number of study units, $S_{jl}$ is the hospital’s service capacity and $D_{r}$ is the search radius. Each level has three search radius values. In addition, $d_{kj}$ represents the distance between a residential area and a hospital, and $P_{k}$ is the population of each study unit.

(2) Calculating the spatial access in each study unit

To consider the effect of distance decay on search scope, a weight that is inversely proportional to the distance can be calculated according to the distance between residential areas and hospitals. The spatial access in each study unit, $A_{k}$, can be indicated by the following formula:

(5) $$A_{k}=\lambda \cdot \sum_{k\in \{d_{kj}\in D_{r}\}}R_{jl}\cdot \frac{1}{d_{kj}}(l=A,B,C)$$

where l is the hospital level, j is the number of hospitals at that same level, k is the number of study units, $S_{jl}$ is the hospital’s service capacity and $D_{r}$ is the search radius. Each level has three search radius values. In addition, $d_{kj}$ represents the distance between a residential area and a hospital, and λ is the hospital level.

## 3. Results and Discussion

### 3.1. Spatial Distribution of Hepatitis B Morbidity in Shenzhen

#### 3.1.1. Hepatitis B Data Processing

**Figure 3:**
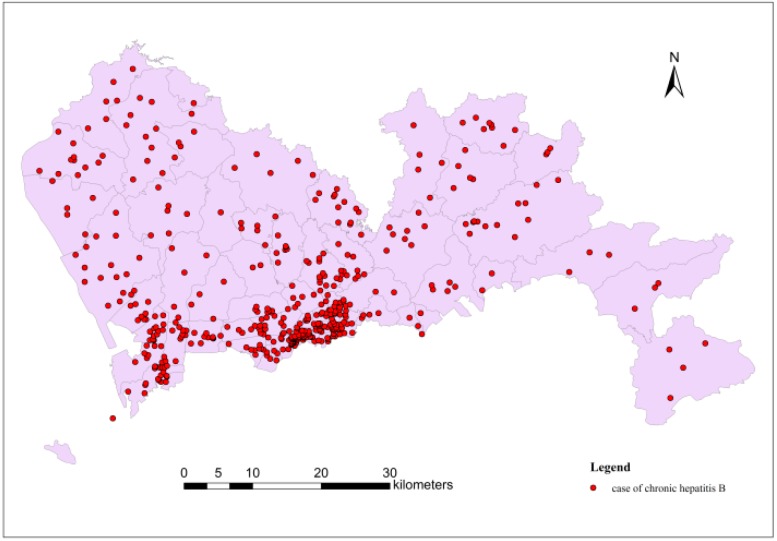
Distribution of hepatitis B cases in Shenzhen in 2010.

The statistical data for this paper included all new hospitalized hepatitis B cases in Shenzhen in 2010. In this group of data, there were 2,851 new hepatitis B cases. As shown in Figure 3, the distribution of hepatitis B cases was uneven, as cases were concentrated in the southwest of Shenzhen, but sparse in the rest of the city.

**Figure 4:**
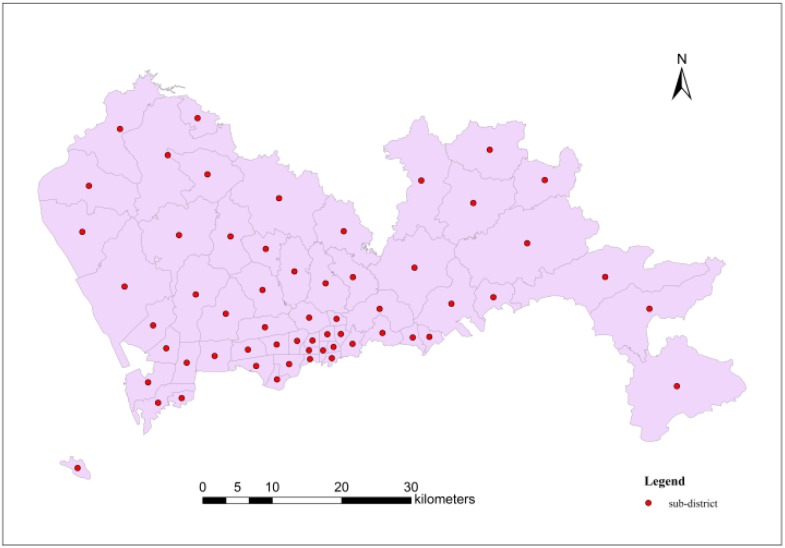
The case data points in each sub-district.

The home addresses of case were accurate to the sub-district level; as a result, geometric centre points of each sub-district could be used to represent respective spatial position (shown in Figure 4).

These points represent the number of hepatitis B cases in each sub-district, as shown in Table 2. According to the table, Nantou, Lianhua and Yuehai demonstrated the most cases in Shenzhen in 2010.

**Table 2:** The number of hepatitis B cases in each sub-district.

Sub-District Name	Number of Cases	Sub-District Name	Number of Cases	Sub-District Name	Number of Cases
Shekou (N)	7	Dalang	7	Huangbei	21
Shekou (S)	39	Haishan	1	Liantang	12
Zhaoshang	17	Shatoujiao	17	Dongxiao	6
Nanshan	9	Meisha	3	Qingshuihe	8
Shahe	21	Yantian	13	Donghu	37
Nantou	1443	Shatou	14	Dapeng	5
Taoyuan	4	Nanyuan	22	Nanwan	22
Xili	39	Huaqiang North	21	Kuiyong	5
Yuehai	74	Xiangmihu	15	Buji	73
Longhua	66	Lianhua	88	Bantian	18
Xixiang	57	Yuanling	10	Henggang	19
Fuyong	9	HuaFu	11	Pingshan	57
Shiyan	74	Meilin	40	Pinghu	48
Shajin	30	Fubao	7	Longgang	7
Guangming (N)	3	Futian	45	Kengzi	5
Guangming (S)	11	Nanhu	29	Pingdi	37
Gongming	50	Guiyuan	17	Longcheng	12
Songgang	38	Dongmen	13	Nan’ao	8
Minzhi	10	Sungang	8	Xin’an	15
Guanlan	39	Cuizhu	15		

The raw statistical data based on the regional administrative units indicated the number of hepatitis B cases. These data were not suitable for spatial interpolation, but they can be converted to morbidity rates in epidemiological studies. The morbidity of hepatitis B was expressed as:

(6) $$M=\frac{N_{c}}{P}$$

where *M* is the morbidity, $N_{c}$ is the number of new cases, and *P* is the exposed population.

In fact, with a larger population in a specific region, the morbidity from this calculation could be more accurate, and with a smaller population, the morbidity might be less accurate; thus, these morbidity data cannot reflect the true spatial distribution of hepatitis B. Based on the population in Shenzhen in 2010, there was a noticeable disparity among the different regions, making it necessary to adjust the morbidity in each sub-district to improve the accuracy.

Given that the true hepatitis B morbidity $\theta_{i}$ in one sub-district i satisfying the normal distribution $N(\mu_{i},\sigma_{i}^{2})$ is a random variable, the hepatitis B morbidity $t_{i}$ obtained by calculation is only one instance of satisfying the normal distribution $N(\mu_{i},\sigma_{i}^{2})$. Thus, more accurate hepatitis B morbidity can be determined using the Bayesian method and the results will be a linear combination of $t_{i}$ and $\mu_{i}$ [38]. The formulas are as follows:

(7) $${\hat{\theta }}_{i}=w_{i}t_{i}+(1-w_{i})\mu_{i}$$

(8) $$w_{i}=\frac{\sigma_{i}^{2}}{\sigma_{i}^{2}+\mu_{i}/n_{i}}$$

where $w_{i}$ represents a weight with a value between 0 and 1, $n_{i}$ is population in each sub-district, $\mu_{i}$ represents mathematical expectation and $\sigma_{i}^{2}$ represents variance of hepatitis B morbidity. The Equations (7) and (8) indicates that lower population leads to lower $w_{i}$, and $\mu_{i}$ accounts for larger proportion of the weight, and ${\hat{\theta }}_{i}$ is close to the $\mu_{i}$. Conversely, ${\hat{\theta }}_{i}$ is close to the $t_{i}$.

The adjusted result of hepatitis B morbidity can be calculated using the Bayesian method with the mathematical expectation $\mu_{i}$ and variance $\sigma_{i}^{2}$ of the normal distribution in each sub-district. The assessment of $\mu_{i}$ and $\sigma_{i}^{2}$ is complex, whereas there is an easy way that the values of $\mu_{i}$ and $\sigma_{i}^{2}$ in each sub-district are all the same to resolve this challenge, and thus $\mu_{i}$ and $\sigma_{i}^{2}$ can be calculated using the number of new hepatitis B cases and the total population in each sub-district. The formulas are as follows:

(9) $$\mu_{i}=\hat{\mu }=\sum y_{i}/\sum n_{i}$$

(10) $$\sigma_{i}^{2}=\frac{\sum n_{i}{\left(t_{i}-\hat{\mu }\right)}^{2}}{\sum n_{i}}-\frac{\hat{\mu }}{\bar{n}}$$

where $\hat{\mu }$ and $\bar{n}$ represents the mean value of hepatitis B morbidity and population in the study area, respectively, and $y_{i}$ is the number of new hepatitis B cases in each sub-district.

**Table 3:** The hepatitis B morbidity in each sub-district.

Sub-District Name		Morbidity (10^4^)		Sub-District Name	Morbidity (10^4^)	Sub-District Name	Morbidity (10^4^)
Shekou (N)		1.56		Dalang	0.29	Huangbei	1.81
Shekou (S)		7.53		Haishan	0.33	Liantang	1.40
Zhaoshang		2.05		Shatoujiao	3.13	Dongxiao	0.67
Nanshan		0.64		Meisha	1.56	Qingshuihe	0.85
Shahe		1.70		Yantian	1.59	Donghu	4.06
Nantou		88.40		Shatou	0.65	Dapeng	1.12
Taoyuan		0.36		Nanyuan	1.97	Nanwan	1.13
Xili		2.04		Huaqiang North	3.49	Kuiyong	0.90
Yuehai		4.49		Xiangmihu	1.65	Buji	2.01
Longhua		1.78		Lianhua	5.01	Bantian	0.84
Xixiang		0.99		Yuanling	1.16	Henggang	0.64
Fuyong		0.22		HuaFu	1.53	Pingshan	2.70
Shiyan		2.92		Meilin	2.32	Pinghu	2.07
Shajin		0.58		Fubao	0.72	Longgang	0.37
Guangming N)		1.36		Futian	1.89	Kengzi	0.61
Guangming (S)		2.28		Nanhu	3.02	Pingdi	3.66
Gongming		1.21		Guiyuan	1.98	Longcheng	0.49
Songgang		0.96		Dongmen	1.43	Nan’ao	3.28
Minzhi		0.39		Sungang	1.27	Xin’an	0.39
Guanlan		0.87		Cuizhu	1.36		

Table 3 shows the adjusted hepatitis B morbidity in each sub-district, which was used for kriging interpolation after preprocessing. Before using kriging interpolation, morbidity data must be converted using a statistical method. Logarithmic conversion is widely used in this field, as it tends to shift data tend to a normal distribution and satisfy the data’s stationary hypothesis [39]. After the conversion, the morbidity data were in accordance with the demand of kriging interpolation.

#### 3.1.2. The Spatial Distribution of Hepatitis B Morbidity

**Figure 5:**
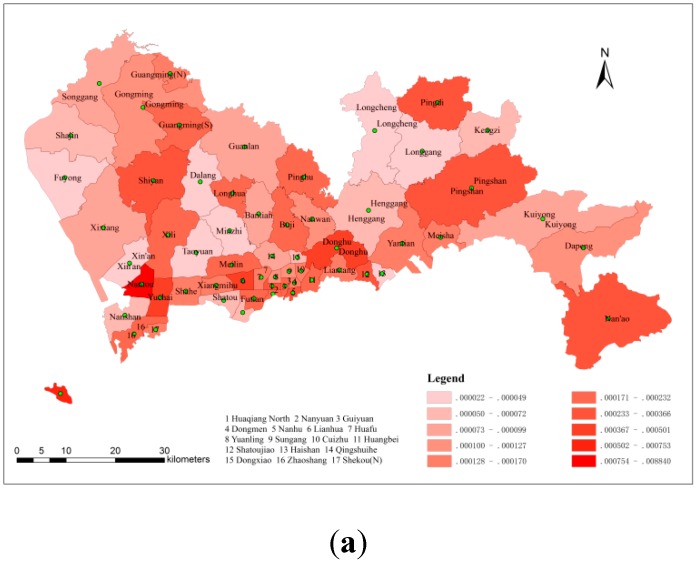

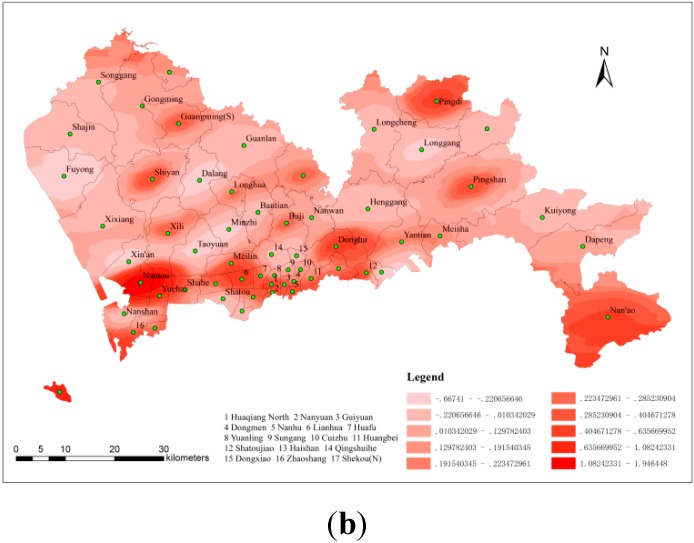
(**a**) The spatial distribution of hepatitis B morbidity (non-interpolated) (**b**) The spatial distribution of hepatitis B morbidity (with kriging).

Figure 5 shows the spatial distribution of hepatitis B morbidity; Figure 5 used no interpolation method, whereas Figure 5b used kriging interpolation. In general, the locations of high morbidity on both graphs were the same, and the highest morbidity was observed in the southwest of Shenzhen. Nantou and its vicinity exhibited the most hepatitis B infections, and Guangming, Pingdi, Shiyan, Pingshan, Lianhua, Donghu, Nan’ao, Shekou (S) and their respective vicinities represented the other high-morbidity areas in Shenzhen in 2010. Comparing these two graphs, Figure 5a shows a statistical map of grade, whereas Figure 5 shows a contour map that weakens the influence of administrative boundaries on the spatial distribution of hepatitis B morbidity. The population often flows constantly from one district to another, and thus the results shown in Figure 5 better reflect the reality.

### 3.2. Spatial Risk Factor Analysis of the Spread of Hepatitis B

#### 3.2.1. The Types of Risk Factors

The kriging interpolation results show that there were areas of high hepatitis B morbidity, and it can be inferred that some risk factors may have had an effect on this high morbidity. Hepatitis B is an infectious disease caused by HBV that is mainly transmitted through blood or body fluids. According to the conclusions of previous studies, some service facilities such as bath centres, beauty salons, massage parlours and pedicure parlours contribute to a high risk for HBV infection [40,41,42,43]. The shared utensils used at these facilities are not only unsterile, but also the skin of customers and service staff members can be easily broken during this services. Therefore, these four types of service facilities can be regarded as dangerous point sources of HBV infection.

#### 3.2.2. Data Processing of Risk Factors

**Figure 6:**
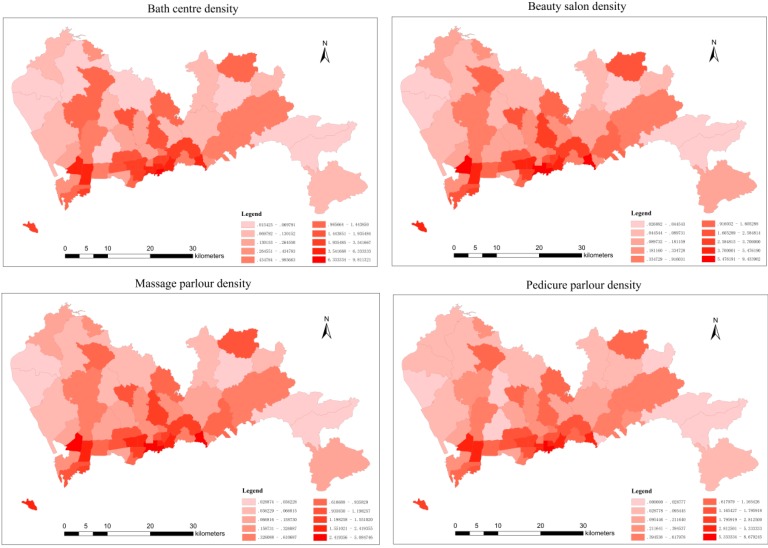
The spatial distribution density of the four types of service facilities.

Data concerning the numbers and addresses for these four types of service facilities were extracted from Baidu maps. As shown in Figure 6, the spatial distribution of the four types of service facilities was uneven, with increasing numbers detected in southwest Shenzhen.

To accurately reflect the true spatial distribution of the four types of facilities, their density in each sub-district could be calculated with the following formula:

(11) $$D_{f}=\frac{N_{f}}{S}$$

where $D_{f}$ is the density of the facilities in each sub-district, $N_{f}$ is the number of each type of facility in each sub-district, and S is the area of each sub-district.

Figure 6 shows the spatial distribution density of each type of service facility in each sub-district. The highest was observed in the south and southwest of Shenzhen. Comparing with Figure 5a,b and Figure 6, it is clear that there was a positive correlation between the hepatitis B morbidity distribution and the density distribution of the four types of service facilities. The correlation between these two factors could be measured accurately by calculating the Pearson correlation coefficient.

#### 3.2.3. The Calculation and Explanation of the Pearson Correlation Coefficient

Table 4 shows the Pearson correlation coefficients calculated between each type of facility and the hepatitis B morbidity after performing a t-test (*p* < 0.05). In general, there was a positive correlation between these types of facilities and the hepatitis B morbidity, which suggests that these facilities have a positive effect on the spread of hepatitis B. Among the Pearson correlation coefficient results, the coefficient between massage parlours and hepatitis B morbidity was the largest of the four types of facilities, followed by bath centres, pedicure parlours and beauty salons.

**Table 4:** Pearson correlation coefficients between hepatitis B morbidity and the type of service facility.

Bath Centres	Beauty Salons	Massage Parlours	Pedicure Parlours
0.566	0.515	0.626	0.538

Interpretation of the Pearson correlation coefficients is shown in Table 1. Because hepatitis B studies are related to the social sciences, the interpretation of coefficients in this table is considered to be reasonable and reliable. From the calculation results, each of the four types of service facilities showed a positive correlation with hepatitis B morbidity in Shenzhen in 2010. Although these four types of facilities to an extent can make people’s life convenient and drive local economic development, they greatly increase the risk of hepatitis B infection. Because of factors such as the large number of HBV-infected individuals in China, the troubling health status in the service facilities and a lack of disease prevention awareness, these four types of service establishment can be easily regarded as dangerous sources of HBV infection. Distinguishing between spatial risk factors not only helps to analyze how they influence the spatial spread of epidemic diseases, but also contributes to establishing disease prevention and control measures.

### 3.3. Analysis of Spatial Access to Medical Resources

#### 3.3.1. The Spatial Distribution of Medical Resources

**Figure 7:**
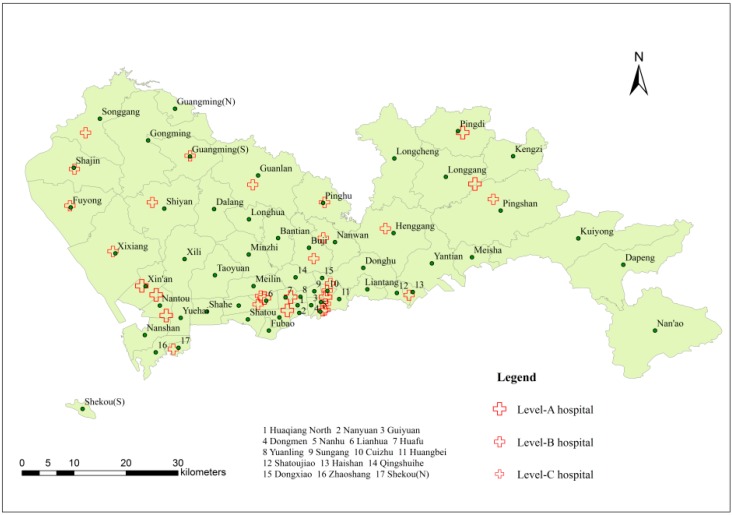
Distribution of liver disease hospitals.

According to statistics from the Shenzhen Centre for Health Information, there were 139 hospitals in Shenzhen in 2010, and 65 hospitals with level certificates were related to the prevention and control of liver diseases. On the basis of the scale and level of medical treatment, the hospitals were divided into three levels in descending order: A, B and C. As shown in Figure 7, in terms of the number of hospitals, most hospitals were located in the southwest or south of Shenzhen. Specifically, there were 9 level-A hospitals, which have the largest red crosses; 22 level-B hospitals, with slightly smaller crosses; and 34 level-C hospitals, with the smallest crosses. Most of the hospitals with high service ability are also located in the southwest or south of Shenzhen. Thus, it can be concluded that Shenzhen lacks high-quality medical resources and that spatial distribution of medical resources is extremely uneven regardless of quantity or quality.

#### 3.3.2. The Calculation of Spatial Access

The spatial access to medical resources was measured using the improved two-step floating catchment area method. The main calculation progress is described below:

1. Setting the value of the search radius

Based on the mean population density of Shenzhen and the number of people the hospitals provide with medical service [44], the search radius values for the different hospital levels were calculated. The result indicated that the level-A hospital search radius was 6 km, the level-B hospital search radius was 3 km, and the level-C hospital search radius was 1.7 km.

To more accurately determine the hospital service radius values, a group of gradient search radius values was set for the same hospital levels. Because Shenzhen lacked medical resources, the search radius value had to be somewhat larger. Therefore, for the level-A hospitals, the gradient search radius values were 6, 9 and 12 km; for the level-B hospitals, they were 3, 4 and 5 km and for the level-C hospitals, they were 1.7, 2 and 2.3 km.

2. Calculating the ratio of supply and demand

The calculation of spatial access to medical resources could be divided into three groups according to the different search radius values. Specifically, values of 6, 3 and 1.7 km, were used for the first group; 9, 4 and 2 km for the second group; and 12, 5 and 2.3 km for the third group.

Using the different search radius values for each group, search circles could be drawn using buffer analysis in ArcGIS Desktop (Environmental Systems Research Institute, Inc. [ERSI], Redlands, CA, USA). The number of points in the search circle was counted and the corresponding points in each sub-district were determined. Finally, each hospital’s supply and demand ratio was calculated using Equation (4).

3. Calculating the spatial access in each sub-district

Equation (5) uses two parameters: the distance between a residential area and a hospital, and each hospital’s level coefficient. The distance between the residential areas and hospitals was measured using the tools in ArcGIS. The hospital level coefficients, which were determined by hospital level, were 3, 2 and 1, corresponding to hospital levels A, B, and C. Then, spatial access to the hospitals in each sub-district could be calculated using Equation (5). However, some sub-districts may not have been included in any search circles; this would indicate that no services could be obtained for these sub-districts, which was highly unlikely. To resolve this challenge, we selected the nearest hospital to the sub-district and calculated its spatial access using the same process. Table 5 shows the spatial access to hospitals in each sub-district within different search radiuses.

**Table 5:** Spatial access to hospitals in each sub-district within different search radiuses.

Sub-District Name	Spatial Access to Hospitals at Different Search Radiuses (10^3^)		
	6, 3, 1.7 km	9, 4, 2 km	12, 5, 2.3 km
Shekou (N)	22.23	26.52	29.97
Shekou (S)	0.74	0.74	0.74
Zhaoshang	6.02	8.35	10.32
Nanshan	4.18	5.60	6.33
Shahe	2.30	5.64	9.26
Nantou	13.92	13.92	13.92
Taoyuan	1.49	2.76	7.24
Xili	0.67	4.09	4.77
Yuehai	7.04	8.44	9.93
Longhua	0.26	0.26	0.62
Xixiang	2.35	2.68	2.91
Fuyong	4.02	4.02	4.02
Shiyan	3.06	3.24	4.31
Shajin	4.35	4.35	4.35
Guangming (N)	3.05	3.05	3.05
Guangming (S)	6.02	6.02	6.02
Gongming	0.19	0.19	0.19
Songgang	0.43	0.43	0.43
Minzhi	0.82	1.48	2.50
Guanlan	0.70	0.70	0.70
Dalang	0.28	0.28	0.28
Haishan	11.34	11.34	13.46
Shatoujiao	5.95	5.95	9.24
Meisha	3.01	3.01	8.03
Yantian	1.08	1.08	1.08
Shatou	4.13	5.82	7.16
Nanyuan	20.63	24.50	25.40
Huaqiang North	51.10	58.86	60.84
Xiangmihu	7.82	14.52	19.33
Lianhua	30.58	31.72	31.72
Yuanling	29.13	35.10	36.31
HuaFu	54.17	57.96	58.23
Meilin	10.12	10.26	11.30
Fubao	9.60	12.30	12.30
Futian	8.31	10.47	10.47
Nanhu	42.56	46.43	47.67
Guiyuan	31.86	36.20	36.20
Dongmen	47.61	49.84	49.84
Sungang	42.51	46.05	47.65
Cuizhu	154.18	155.82	156.69
Huangbei	16.49	20.47	21.82
Liantang	3.51	3.51	7.98
Dongxiao	16.23	21.64	23.67
Qingshuihe	14.37	15.03	17.70
Donghu	0.20	2.76	6.57
Dapeng	0.13	0.13	0.13
Nanwan	1.60	3.22	4.67
Kuiyong	0.62	0.62	0.62
Buji	2.01	3.15	4.10
Bantian	0.38	1.58	2.89
Henggang	1.85	1.85	1.85
Pingshan	2.67	2.67	2.67
Pinghu	11.06	11.06	11.48
Longgang	1.77	2.82	2.82
Kengzi	2.49	4.64	4.64
Pingdi	24.79	26.83	26.83
Longcheng	0.60	0.60	1.11
Nan’ao	0.40	0.40	0.40
Xin’an	10.16	10.16	10.16

4. Kriging interpolation of spatial access

Kriging interpolation can also be used to determine the spatial access to medical resources. Using the same method of logarithmic conversion, the spatial access data for each sub-district were transformed to approximate the normal distribution and satisfy the stationary data hypothesis.

#### 3.3.3. The Distribution of Spatial Access 

**Figure 8:**
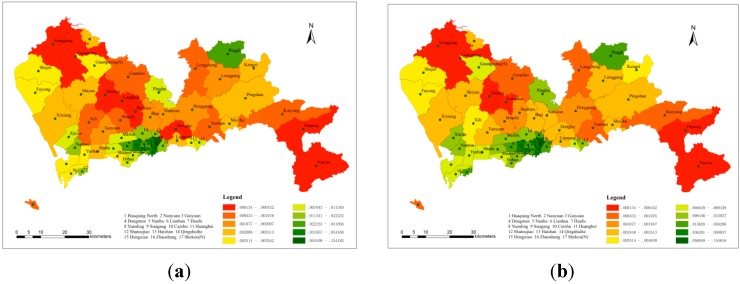

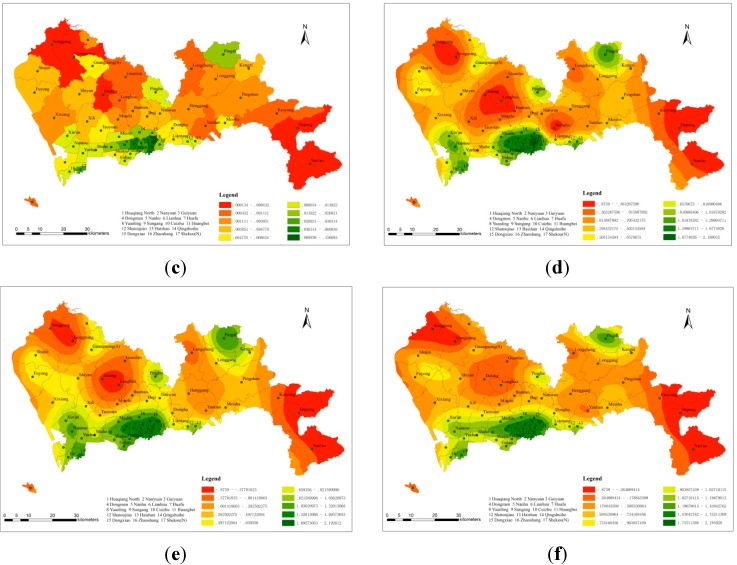
The spatial access in each sub-district. (**a**) 6, 3, 1.7 km (non-interpolated); (**b**) 9, 4, 2 km (non-interpolated); (**c**) 12, 5, 2.3 km (non-interpolated); (**d**) 6, 3, 1.7 km (with kriging); (**e**) 9, 4, 2 km (with kriging); (**f**) 12, 5, 2.3 km (with kriging).

Figure 8a–c shows the spatial access in each sub-district, without interpolation, in the three different search radius groups. The search radius values in Figure 8a were 6, 3 and 1.7 km, corresponding to hospital levels A, B, and C, respectively. Similarly, the values in Figure 8b,c were 9, 4, 2 km and 12, 5, 2.3 km, respectively. These values showed the same corresponding relationship with hospital level. Within the three groups, Cuizhu, Huaqiang North and Huafu were the sub-districts with the most spatial access. Figure 8d–f shows three contour maps, for which kriging interpolation was used, that were also based on the different search radius values. The locations of high and low spatial access were approximately the same, and the distribution of access was uneven as well. In general, there were three centres with high spatial access, which were concentrated in the southwest, south and northeast, and two low-access centres, which were concentrated in the northwest and southeast. The reason for this distribution pattern is that most of the high-quality medical resources are concentrated in the southern area, which is the economic centre of Shenzhen. In addition, most of the level-A hospitals which have sufficient paramedics, advanced medical facilities, and professional medical staff, are located in the same region, whereas in areas with low spatial access, there is a lack of medical resources to meet the demands of the surrounding population.

#### 3.3.4. Relationship between Hepatitis B Morbidity and Spatial Access

**Figure 9:**
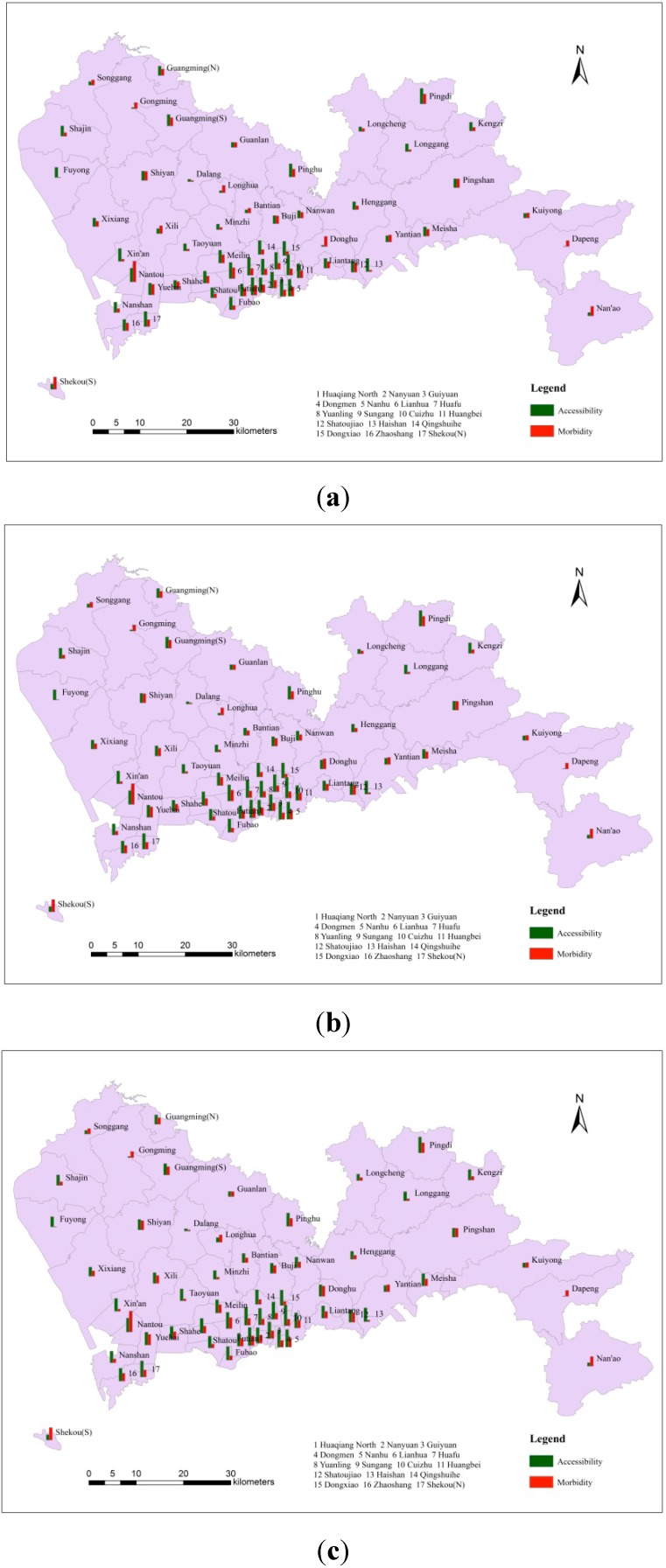
The comparison between spatial access and hepatitis B morbidity. (**a**) Search radiuses: 6, 3, 1.7 km; (**b**) Search radiuses: 9, 4, 2 km; (**c**) Search radiuses: 12, 5, 2.3 km.

The adequacy of medical resources can be measured by the spatial access index, and comparing a patient’s regional access to medical resources with disease morbidity make it possible to determine whether the regional medical resource distribution is adequate for this particular disease. In Figure 9, the green column represents access to regional medical resources, and the orange column represents hepatitis B morbidity. It can be seen from these three figures that regardless of the search radius, in sub-districts with high hepatitis B morbidity, the spatial access index was generally higher; whereas in sub-districts with low morbidity, the spatial access index was generally lower. This result indicated that hospitals were able to provide sufficient medical services for neighbouring hepatitis B patients. In general, the allocation and spatial distribution of medical resources for hepatitis B were adequate.

## 4. Conclusions

1. In epidemiological research, mining geographic spatial data can not only reveal the spatial distribution characteristics of infectious diseases but also may identify risk factors that have a major impact on the spread of these diseases. In addition, assessing the spatial access to medical resources can reveal the accuracy of medical resource spatial distribution. These studies have important significance for establishing infectious disease prevention and control measures.

2. The spatial distribution of hepatitis B; the distribution of high-transmission-risk facilities such as bath centres, beauty salons, massage parlours and pedicure parlours and access to regional medical resources were uneven in Shenzhen in 2010, with a main concentration of such factors in the south and southwest, which correspond to the economic centre regions of Shenzhen. On the one hand, these distribution characteristics indicated that hepatitis B infection had a positive correlation with the four high-risk service facilities. On the other hand, the allocation and spatial distribution of medical resources for hepatitis B was adequate.

3. In view of these conditions, measures must be taken to strengthen the prevention and control of hepatitis B, such as improving the sanitary conditions in service facilities, raising awareness about the spread and prevention of hepatitis B and strengthening the construction of medical facilities in economically less-developed areas in Shenzhen.

4. This paper did not research spatial distribution of hepatitis B in a temporal manner because of limited data. In addition, the correlation between hepatitis B and high-risk locations was not very strong, and thus further studies are necessary to determine whether hepatitis B correlates with certain geographical or other elements in Shenzhen city. Furthermore, The method, the improved two-step floating catchment area model to evaluate the spatial access to medical resources must continuously be modified for more accurate evaluation results. These limitations will be studied further in the future.
